# A modified mTNM staging system based on lymph node ratio for colon neuroendocrine tumors: A recursive partitioning analysis

**DOI:** 10.3389/fsurg.2022.961982

**Published:** 2022-10-21

**Authors:** Ye Wang, Huajun Cai, Yiyi Zhang, Jinfu Zhuang, Xing Liu, Guoxian Guan

**Affiliations:** ^1^ Department of Colorectal Surgery, The First Affiliated Hospital of Fujian Medical University, Fuzhou, China; ^2^ Department of Colorectal Surgery, Fujian Medical University Union Hospital, Fuzhou, China

**Keywords:** prognosis, lymph node ratio, recursive partitioning analysis, neuroendcrine tumor, colon

## Abstract

**Background:**

In the current tumor–lymph node–metastasis (TNM) staging system for colon neuroendocrine tumors, lymph node status is divided into N1 and N0. An assessment of the lymph node ratio (LNR) and a proposal for a modified mTNM staging system were the objectives of this study.

**Methods:**

Selecting the optimal cut-off value of LNR was done using X-tile. A Cox regression model and the Kaplan–Meier method were performed to calculate patient cancer-specific survival in the Surveillance, Epidemiology and End Results cohort. Recursive partitioning analysis was used to improve TNM staging.

**Results:**

The study included 674 patients. The current TNM staging system showed inadequate discriminatory power between stage I and stage II patients (*p* = 0.088). The optimal cut-off value was determined as 0.6 for LNR. Based on multivariate Cox regression analysis, the modified mN classification could be classified into mN 0 (LNR = 0.00), mN 1 (LNR = 0.01–0.60), and mN 2 (LNR > 0.60), and was found to be an independent factor affecting prognosis (*p* < 0.001). Using the American Joint Committee on Cancer T and modified mN classifications, the modified mTNM system was constructed, and it exhibited better prognostic discriminatory power ability than the traditional TNM system (C-index: 0.587 vs. 0.665).

**Conclusions:**

Our study determined that LNR is a prognostic factor in colon NET patients. In addition, to more accurately assess the prognosis of colon NET patients, we proposed a modified mTNM staging system.

## Introduction

The number of neuroendocrine tumors (NETs) has increased steadily over the past 40 years to 5 per 100,000 people (1–3). Colon NETs account for 7.8% of all NETs (4) and carry a significantly worse survival among all NETs (5). Considering the increasing incidence and the poor prognosis of colon NETs, prognostication of colon NETs have become imperative to tailor postoperative therapy and surveillance to individual patient needs.

Currently, among the most widely used prognostic evaluation systems for colon NETs is the tumor–lymph node–metastasis (TNM) classification proposed by the American Joint Committee on Cancer (AJCC) (6). Lymph node (LN) status is an important indicator for the prognosis of colon NETs (7). In the 2017 eighth edition AJCC staging manual, N0 (no nodal metastasis in regional lymph nodes) and N1 (no regional metastasis in regional lymph nodes) are the only criteria for defining lymph node metastasis (8), which are in accordance with that of the North American Neuroendocrine Tumor Society (NANETS) (4) and the European Neuroendocrine Tumor Society (ENETS) (9). However, the classification of lymph node metastases was independent of the number of positive lymph nodes. At present, some studies have shown that the number of positive LNs in the gastroenteropancreatic NETs is of great significance for judging prognosis (10, 11). The prognostic significance of the ratio of positive LNs to the total number of retrieved LNs (LNR) has been rigorously corroborated in pancreatic and small intestinal NETs, owing to the advantage over the simple binary classification of LN metastasis (N0 or N1) (12, 13). We speculate that the TNM staging system with the addition of LNR will have better prognostic value. However, the effect of LNR on the prognosis of colon NETs has not been investigated yet.

Using LNR for colon NETs as a prognostic factor, this study proposes a novel staging system based on modified mN staging.

## Materials and methods

### Patient population

The population-based analysis was based on data from the National Cancer Institute's Surveillance, Epidemiology and End Results (SEER) program. The SEER records collect a variety of information about cancer patients, including surgical approach and extent of lymph node resection (14). The site and morphology of the collaborative phase data acquisition system (CS Schema v0204+) were used to select patients with colon NETs from 1988 to 2016. The histological type and tumor location were classified using the following code: NET Colon (15). Our study was based on the following inclusion criteria: (1) patients over 18 years of age; (2) patient survival >1 month; (3) the number of retrieved LNs ≥1. We excluded patients if they had a distant metastasis at diagnosis or unconfirmed lymph node and T stage information. Finally, there were 674 patients with colon NETs enrolled in the study. Baseline clinicopathologic characteristics, including age, gender, race, grade, TN stage, number of retrieved LNs, and number of positive LNs, and SEER data were used to compile this study. The ratio of the number of positive LNs to the total number of dissected LNs was defined as LNR.

### Statistical analysis

All statistical analyses were done by SPSS statistical package (version 23; IBM, Armonk, NY, United States). Categorical variables were compared by the Chi-square test, while continuous variables were evaluated using two-sample independent *t*-test or Wilcoxon rank-sum tests. The primary endpoint was cancer-specific survival (CSS), which is calculated as the time interval from diagnosis to death or last follow-up. Survival outcomes were assessed using the log-rank test and the Kaplan–Meier method. Univariate analyses' significant variables (*p* < 0.05) were incorporated into a Cox proportional regression to find independent risk factors for CSS. The X-tile algorithm was used to determine the best cut-off for LNR and LNs (Version 3.6.1, Yale University) using the minimum *p* values from the log-rank *χ*^2^ statistics. The X-tile plot used two-dimensional projections of different cut-off points to show survival outcomes in relation to different lymph node ratios. The optimal cut-off point of LNR was determined according to the minimum *P*-value obtained by log-rank χ^2^ analysis. Age, tumor grade, and the AJCC T classification LNR or AJCC N classification were used to establish the predictive model. Through recursive partitioning, patients are divided into different risk groups. The recursive partitioning analysis (RPA) method is a method used to statistically analyze multivariate data. RPA separates predictors into different groups by establishing a decision tree that can divide patients into homogeneous risk groups. The best split for each variable within each node was examined, as well as the optimal split corresponding to that split, which has the greatest difference between patient groups (16). RPA was implemented using the R package “rpart.” *p* < 0.05 on both sides was considered statistically significant. Model comparisons were made based on the C-index. Higher C-index indicates a higher degree of accuracy in prognostication. Since the data are from the SEER database, patient consent was not required.

## Results

### Patient population

A total of 674 patients with colon NETs between 1988 and 2016 from the SEER database were eligible for this analysis. The median age was 66 years. Among them, 202 patients were included in the AJCC N0 group and 472 patients in the AJCC N1 group. Age, gender, and race of the two groups did not differ statistically significantly (all *p* > 0.05) in Supplementary Table S1. Compared with the AJCC N0 group, patients with AJCC N1 stage had higher T stage and worse differentiation.

### Identification of predictors of CSS based on TNM staging system

**Figure 1:**
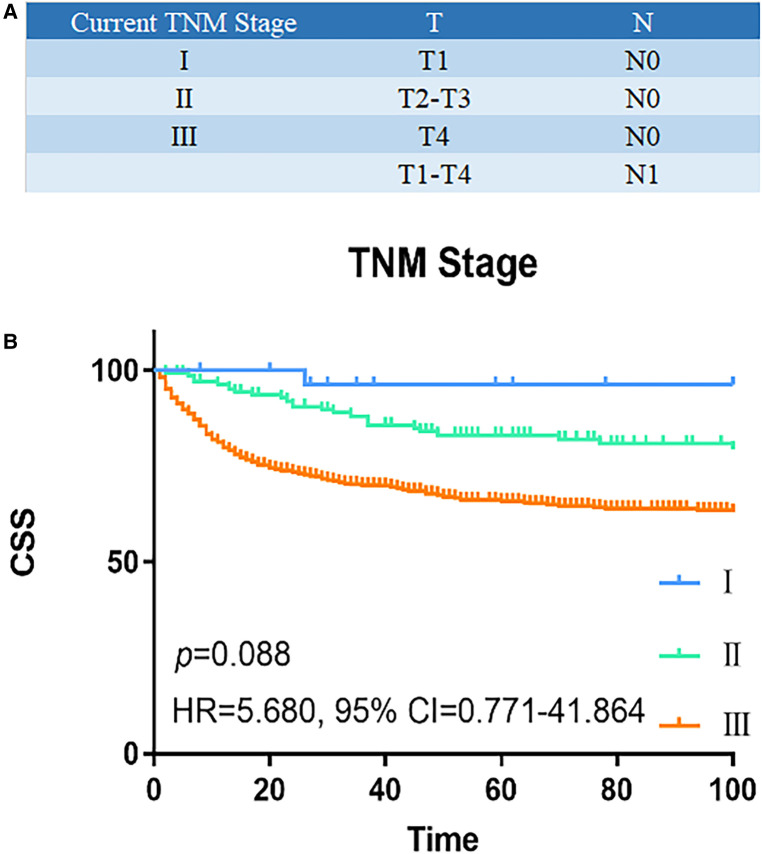
Survival analysis of current TNM staging system. (**A**) Current TNM staging system: I (T1N0M0), II (T2-3N0M0), III (T4N0M, TxN1M0), and IV (TxNxM1). (**B**) The CSS curve based on TNM staging system. The survival curves of stage I and II patients overlapped (HR, 5.680, 95% CI, 0.771–41.864, *p* = 0.088). *P* values are derived from the Cox risk model. CSS, cancer-specific survival; TNM, tumor–lymph node–metastasis; HR, hazard ratio; CI, confidence interval.

Univariate and multivariate Cox regression analyses were used to determine predictive factors of CSS based on the TNM staging system in Table 1. In univariate analysis, older age [≥66 years, hazard ratio (HR), 2.185, 95% confidence interval (CI), 1.638–2.915, *p* < 0.001], male (HR, 0.752, 95% CI, 0.568–0.995, *p* = 0.046), tumor differentiation (poorly/undifferentiated, HR, 3.326, 95% CI, 2.308–4.793, *p* < 0.001), higher AJCC T classification (T stage4, HR, 4.937, 95% CI, 2.255–10.807, *p* < 0.001), higher AJCC N stage (N1, HR,1.790, 95% CI, 1.283–2.498, *p* = 0.001), receive chemotherapy (Yes, HR, 1.790, 95% CI, 1.283–2.186, *p* < 0.001), and more advanced TNM stage (stage III, HR, 12.955, 95% CI, 1.815–92.481, *p* = 0.011) were associated with a worse CSS. In a multivariate analysis, older age (≥66 years, HR, 2.006, 95% CI, 1.490–2.701, *p* = 0.001), tumor differentiation (poorly/undifferentiated, HR, 2.354, 95% CI, 1.585–3.496, *p* < 0.001), and higher AJCC N stage (N1, HR, 1.836, 95% CI, 1.354–2.491, *p* < 0.001) remained to be independent prognostic indicators for CSS. However, the TNM stage was not independently associated with CSS. Figure 1B showed the CSS curve using the current TNM stage; the survival curves of patients with stage I and stage II disease overlapped (HR, 5.680, 95% CI, 0.771–41.864, *p* = 0.088), indicating poor discrimination between stage I and stage II patients with colon NETs using the TNM system.

**Table 1:** Univariate and multivariate analysis of CSS in colon NETs in the SEER cohort.

Characteristic	*N* (%)	Univariate analysis^a^		Multivariate analysis^a^	
		HR (95% CI)	*p-*value	HR (95% CI)	*p-*value
Age, median	66 (range 22–93) years				
	<66 years	1		1	
	≥66 years	2.185 (1.638–2.915)	**<0.001**	2.006 (1.490–2.701)	**0.001**
Gender					
Male	302 (44.8)	0.752 (0.568–0.995)	**0.046**	0.961 (0.736–1.254)	0.769
Female	372 (55.2)	1		1	
Race			**0.026**		0.639
White	553 (82.1)	1		1	
Black	89 (13.2)	0.716 (0.455–1.126)	0.148	0.820 (0.674–1.340)	0.428
Others	32 (5.7)	1.783 (1.051–3.023)	0.032	1.132 (0.674–1.901)	0.640
Grade		—			
Well/moderately	128 (19.0)	1	—	1	
Poorly/undifferentiated	337 (50.0)	3.326 (2.308–4.793)	**<0.001**	2.354 (1.585–3.496)	**<0.001**
Unknown	209 (31.0)	—	—		
T classification^b^		—	**<0.001**		**0.008**
T1	56 (8.3)	1			1
T2	76 (11.3)	0.831 (0.301–2.292)	0.721	0.646 (0.289–1.444)	0.287
T3	412 (61.1)	2.834 (1.325–6.602)	0.007	1.290 (0.709–2.346)	0.405
T4	130 (19.3)	4.937 (2.255–10.807)	<0.001	1.792 (0.945–3.399)	0.074
N classification^b^		—			
N0	202 (30.0)	1		1	
N1	472 (70.0)	1.790 (1.283–2.498)	**0.001**	1.836 (1.354–2.491)	**<0.001**
Chemotherapy					
No	546 (81.0)	1		1	
Yes	128 (19.0)	1.701 (1.324–2.186)	**<0.001**	1.165 (0.867–1.566)	0.311
TNM stage^b^		—	**<0.001**		
I	29 (4.3)	1			
II	142 (21.1)	5.680 (0.771–41.864)	0.088		
III	503 (74.6)	12.955 (1.815–92.481)	0.011		

NETs, neuroendocrine tumors; CSS, cancer-specific survival; HR, hazard ratio; CI, confidence interval; SEER, Surveillance, Epidemiology and End Results; TNM, tumor–lymph node–metastasis.

*p* values less than 0.05 are marked as bold.

^a^ Univariate and multivariate analyses were conducted using Cox proportional hazards regression model.

^b^ Staging based on current eighth AJCC system.

### Development of the new nodal stage based on LNR

**Figure 2:**
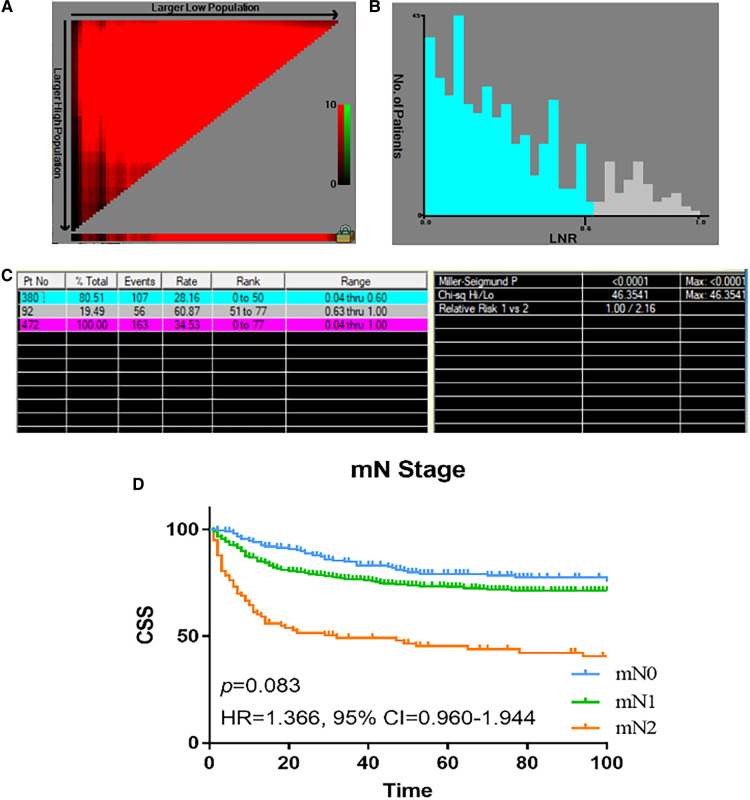
The optimal cut-off value of LNR in patients with colon NETs in the SEER registry was obtained by X-tile analysis. (**A**, **B**) The optimal cut-off value of LNR points of colon NETs patients. X-tile curve for number of LNR established by patients with colon NETs. (**C**) X-tile i mages of LNR and CSS after radical resection of colon NETs. (**D**) Analyses of the survival of mN staging. LNR, lymph node ratio; NETs, neuroendocrine tumors; SEER, Surveillance, Epidemiology, and End Results.

Instead of using a binary classification of LN metastasis (N0 or N1), we proposed a new node classification system based on LNR. The best cut-off value of LNR was identified as 0.6 by using X-tile analysis, and the maximum *χ*^2^ value is 46.3541. The relative hazard ratio is 1:2.16, as shown in Figures 2A–C. Accordingly, we proposed a new nodal stage: LNR 0.00 (mN 0), LNR 0.01–0.60 (mN 1), and LNR >0.60 (mN 2). We further compare the characteristics between the mN 0, 1 and 2 groups, as seen in Table 2. Patients in the mN2 group were associated with poorer tumor differentiation and higher AJCC T classification (*p* < 0.001, respectively). According to the Kaplan–Meier curves, the new mN stages could effectively discriminate patients with different CSS (*p* < 0.001) The survival curves of patients with stage I and II disease overlapped (HR = 1.366, 95% CI = 0.960–1.944, *p* = 0.083) (Figure 2D). In addition, multivariate Cox regression analysis of predictors of CSS including the revised mN classification demonstrated that the revised mN classification is an independent predictor of CSS (mN1, HR = 1.628, 95% CI = 1.186–2.236, *p* = 0.003; mN2, HR = 2.591, 95% CI = 1.977–4.407, *p* < 0.001), as shown in Table 3.

**Table 2:** Patient characteristics for proposed mN0/mN1/mN2 stage for colon NETs patients in the SEER cohort.

Characteristics	N0 (*n* = 202)	mN1 (*n* = 374)	mN2 (*n* = 98)	*p-*value
Age				
<66	92 (45.5%)	192 (51.3%)	44 (44.9%)	0.299
≥66	110 (54.5%)	182 (48.7%)	54 (55.1%)	
Gender				0.406
Male	116 (57.4%)	198 (52.9%)	58 (59.2%)	
Female	86 (42.6%)	176 (47.1%)	40 (40.8%)	
Race				0.855
White	168 (83.2%)	305 (81.6%)	80 (81.6%)	
Black	24 (11.97%)	50 (13.4%)	15 (15.3%)	
Others	10 (5.0%)	19 (5.1%)	3 (3.1%)	
Grade				**0.001**
Well	24 (11.9%)	93 (24.9%)	11 (11.2%)	
Moderately	21 (10.4%)	47 (12.6%)	12 (12.2%)	
Poorly	79 (39.1%)	91 (24.3%)	31 (31.6%)	
Undifferentiated	14 (6.9%)	31 (8.3%)	11 (11.2%)	
Unknown	64 (31.7%)	112 (29.9%)	33 (33.7%)	
T classification				**<0.001**
T1	32 (15.8%)	23 (6.1%)	1 (1.0%)	
T2	26 (12.9%)	45 (12.0%)	5 (5.1%)	
T3	113 (55.9%)	234 (62.6%)	65 (66.3%)	
T4	31 (15.3%)	72 (19.3%)	27 (27.6%)	
No. of positive LNs (median, range)	0	2 (1–28)	2 (1–30)	**<0.001**
No. of examined LNs (median, range)	13 (1–56)	13 (2–63)	13 (1–31)	**<0.001**
Chemotherapy				**0.011**
No	170 (84.2%)	311 (83.2%)	65 (66.3%)	
Yes	32 (15.2%)	63 (16.8%)	33 (33.7%)	

NETs, neuroendocrine tumors; SEER, Surveillance, Epidemiology, and End Results; LNs, lymph nodes.

*p* values less than 0.05 are marked as bold.

**Table 3:** Multivariate Cox regression analysis of predictors of CSS based on revised T/mN classification.

Characteristic	Multivariate Cox regression analysis	
	HR (95% CI)	*p-*value
Age, years		
<66	1	**<0.001**
≥66	1.992 (1.480–2.681)	
Gender		0.694
Male	0.948 (0.726–1.238)	
Female	1	
Race		0.534
White	1	
Black	0.772 (0.473–1.262)	0.302
Others	1.102 (0.654–1.857)	0.715
Grade		
Well/moderately	1	
Poorly/undifferentiated	2.299 (1.549–3.411)	**<0.001**
T classification^a^		**0.017**
T1	1	
T2	0.620 (0.277–1.386)	0.244
T3	1.210 (0.663–2.207)	0.535
T4	1.635 (0.858–3.114)	0.135
mN classification^b^		**<0.001**
mN0	1	
mN1	1.628 (1.186–2.236)	0.003
mN2	2.591 (1.977–4.407)	<0.001
Chemotherapy		
No	1	
Yes	1.143 (0.850–1.537)	0.376

CSS, cancer-specific survival; HR, hazard ratio; LNR, lymph node ratio; CI, confidence interval.

*p* values less than 0.05 are marked as bold.

^a^ Staging based on current eighth AJCC system.

^b^ The mN status was based on LNR: LNR 0% (mN 0), LNR 0.01–0.60 (mN 1), and LNR >0.60 (mN 2).

### Modified mTNM staging system

**Figure 3:**
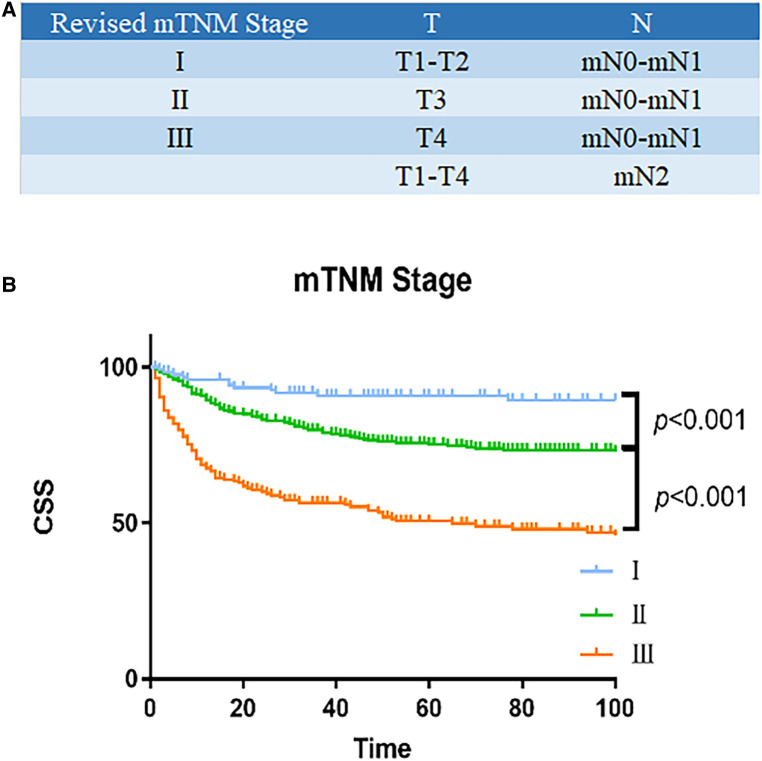
Survival analysis of revised mTNM staging system. (**A**) Modified mTNM. (**B**) Analyses of the survival of mN classification.

**Figure 4:**
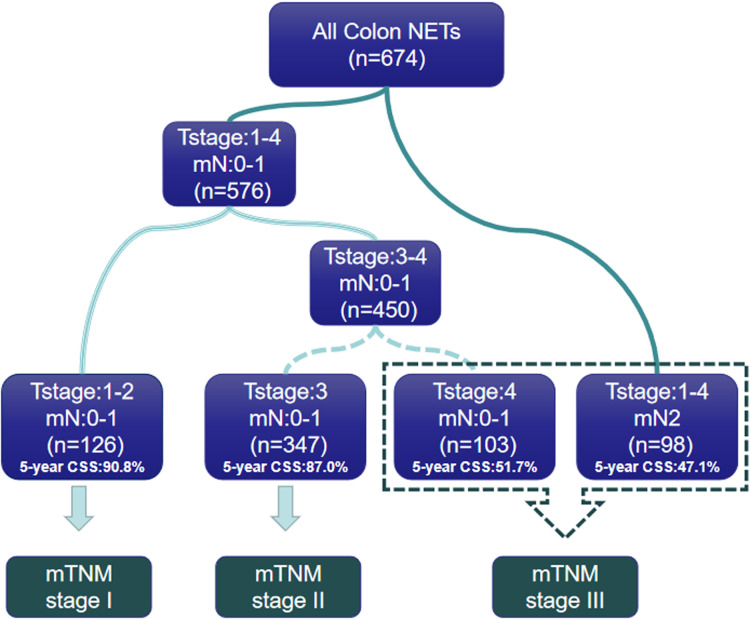
The proposed risk grouping derived by RPA. RPA, recursive partitioning analysis.

In order to propose a new staging system for mTNM, recursive partitioning analysis was performed, as shown in Figures 3A, 4. RPA classified patients into four distinct groups: mTNM I (T1–2 mN0-1), mTNM II (T3 mN0-1), and mTNM III (T4 mN0-1; T1-4 mN 2). As seen in the survival curve (Figure 3B), the revised mTNM staging system is more advantageous than the current staging in distinguishing stage I–III (II-stage: HR, 2.720, 95% CI, 1.520–4.866, *p* = 0.001; III-stage: HR, 7.096, 95% CI, 3.983–12.640, *p* < 0.001).

### Comparison of the predictive models

The higher the index, the high *r* the forecasting accuracy of the forecasting system. The C-index of mN stage is 0.623, and 95% CI is 0.605–0.641, higher than that of the AJCC N stage (0.568 95% CI: 0.554–0.582), indicating a better predictive accuracy. In addition, As measured by prognostic discrimination, the modified TNM staging system performed better than the AJCC TNM staging system (C-index: 0.665 vs. 0.587) as shown in Supplementary Table S2.

## Discussion

The incidence of colonic NETs has continued to increase in recent years. Compared with small intestinal, rectal, gastric, and appendix neuroendocrine tumors, colon neuroendocrine tumors have a worse prognosis (17, 18). In this study, based on the SEER cohort, LNR can be used as a marker to predict the prognosis of patients with colon NETs for the first time and we propose a modified TNM staging system to better assess the prognosis of colon NET patients.

Nowadays, the TNM staging system is the most powerful tool for prognostication of patients with NETs. However, there have only been a few studies that have verified TNM staging system's predictive accuracy for colon NETs. Jann et al. (19) found that the TNM staging system cannot distinguish the survival of stages I/II patients with colon NETs. Similarly, our population-based results indicated that the current AJCC TNM staging system could not discriminate against the survival of stage I from stage II patients. The study by Gong et al. (20) on the eighth edition of AJCC classification standard for colorectal carcinoid cancer found that stage I patients have a better prognosis, stage IV patients have significantly worse prognosis, and the survival outcomes of stage II and stage III patients did not show a significant difference. Therefore, the lack of adequate discriminatory power highlighted the need for modification of the current TNM system.

It has been proven that the AJCC T classification is an accurate predictor of colon NETs (6), which was also ascertained in the current study. However, the predictive accuracy of the AJCC N classification is not satisfactory. The AJCC N classification is determined by whether or not there are positive LNs (N1/N0) (7) and may prevent the TNM staging system from accurately predicting the prognosis of patients with N1 stage. Dasari et al. (3) reported the prognostic importance of positive LNs number in patients with colon NETs and suggested classifying N classification as no positive lymph node, 1 LN metastasis, 2–9 LNs metastasis, and 10 or more positive LNs metastasis. Several articles have raised concerns about the ratio of positive lymph nodes to detected lymph nodes in gastrointestinal tumors; since lymph nodes are often replaced by matted and completely in NET, it is not easy to obtain accurate counts pathologically. In addition, LNR included the number of dissected lymph nodes and adjusted the adequacy of resection to a certain extent. LNR can minimize staged migration. LNR can fully consider the adequacy of surgical lymph node resection and pathological examination of cancer (21). To date, the AJCC proposed T classification in conjunction with LNR classification has been shown to be more effective than the traditional TNM staging system in predicting prognoses in colorectal adenocarcinoma (22–24). However, LNR is still inconclusive as a predictor of NET prognosis. Martin et al. (10) and Petrelli et al. (25) have demonstrated that LNR could predict prognosis in NETs, and can provide a more accurate prognostic assessment. On the contrary, Flatow et al. (26) reported that LNR provided no additional prognostic effect on survival than N0/N1. In their study, only 72 cases of hindgut tumors were included. In addition, they also did not choose a suitable cut-off for LNR. In the present study, 674 patients with colon NETs were included from 1988 to 2016, and the results revealed that higher LNR was associated with poorer survival. Given the large sample size of our SEER cohort, the conclusion might be more rational than previously reported. With the increasing standardization of complete mesocolic excision (CME) surgery for colon cancer and the increasing experience of pathologists, it is important to consider the LNR considering the number of nodes detected.

The cut-off values of LNR varied from 0.2 to 0.5 in previous studies (6, 10, 27); however, these cut-off values were selected based on the experience in other types of NETs. Herein, a cut-off value of 0.6 was established by using X-tile analysis on survival, and three groups of modified mN classifications were determined: mN0, mN1, and mN2. There was no significant difference in survival between mN0 and mN1 patients, suggesting similar survival between N0 and lower LNR patients with colon NETs. It also indicated that the traditional N0/N1 classification cannot accurately evaluate the prognosis, reducing the predictive accuracy of the traditional TNM staging system.

Currently, neither the ENETS nor the NCCN recommend a minimum number of lymph nodes to be retrieved. In colorectal adenocarcinoma, at least retrieval of 12 LNs has become the standard of care (28, 29). We further evaluated the appropriate number of LNs harvested in the colon NETs (Supplementary Figure S1). The optimal number of LNs harvested was 7, reflecting the difference in LNs metastasis between colon NETs and colon adenocarcinoma. To identify the best number of lymph nodes to examine for colon NETs, more research is needed.

According to the AJCC TNM staging system, the presence of lymph node metastases should be classified as stage III, which would obviously underestimate the survival of patients with low LNR. Our modified LNR-based mN classification can further distinguish patients with LN metastasis into high-risk and low-risk groups. Our next task was to compare the predictive accuracy of the proposed mTNM staging system with the AJCC TNM system. The mTNM staging system using LNR showed better performance than the current TNM staging, as indicated by a higher C-index. We have only modified stages I–III, and phase IV remained the same as the current TNM staging system. RPA demonstrated the significant differences of CSS between stage I and stage II patients, reflecting the advantage over the current TNM staging system.

Our research has several advantages. First, the SEER database is a large database that is considered a cluster with relatively strict quality control. Second, this is the first study of modified mN classification based on LNR for colon NETs. Third, a novel mTNM staging system was proposed based on tumor classification and the modified mN classification. Our study does have some limitations. First, this study is a retrospective one, limiting the statistical power of our results to some degree. Second, the SEER database primarily reflects data from western patients; further studies with a larger scale design are warranted before the general applicability of the TNM staging system.

As a result, we found the LNR to be prognostic in patients with colon NETs using the SEER cohort. In addition, we proposed a modified TNM staging system used for more accurately evaluating the prognosis of colon NETs patients.

## Data Availability

The original contributions presented in the study are included in the article/Supplementary Material, further inquiries can be directed to the corresponding author.
