# Supplementary material for: A modified mTNM staging system based on lymph node ratio for colon neuroendocrine tumors: A recursive partitioning analysis
Source: Front Surg. 2022 Oct 21;9:961982. doi: 10.3389/fsurg.2022.961982 (PMC9634476; doi:10.3389/fsurg.2022.961982)
Supplement: Supplementary file 1 [file Table1.docx]

**Supplementary Table S1.** Patient characteristics for AJCC 8th edition N0/N1patients with colon NETs in SEER database

| Characteristic | AJCC N0  (n=202) | AJCC N1  (n=472) | P value |
| --- | --- | --- | --- |
| Age |  |  |  |
| <66 | 82 (45.5%) | 236 (50.0%) | 0.289 |
| ≥66 | 110 (54.5%) | 236 (50.0%) |  |
| Gender |  |  |  |
| Male | 86 (42.6%) | 216 (45.8%) | 0.446 |
| Female | 116 (57.4%) | 256 (54.2%) |  |
| Race |  |  |  |
| White | 168 (83.2%) | 385 (81.6%) | 0.591 |
| Black | 24 (11.9%) | 65 (13.8%) |  |
| Others | 10 (5.0%) | 22 (4.7%) |  |
| Grade |  |  |  |
| Well | 24 (11.9%) | 104 (22.0%) | **0.002** |
| Moderately | 21 (10.4%) | 59 (12.5%) |  |
| Poorly | 79 (39.1%) | 122 (25.8%) |  |
| Undifferentiated | 14 (6.9%) | 42 (8.9%) |  |
| Unknown | 64 (31.7%) | 145 (30.7%) |  |
| T classification |  |  | **<0.001** |
| T1 | 32 (15.8%) | 24 (5.1%) |  |
| T2 | 26 (12.9%) | 50 (10.6%) |  |
| T3 | 113 (55.9%) | 299(61.1%) |  |
| T4 | 31 (15.3%) | 99 (21.0%) |  |
| No. of positive LNs  (median, range) | 0 (0-0) | 2 (1-30) | **<0.001** |
| No. of examined LNs  (median, range) | 13 (1-56) | 13 (1-63) | **<0.001** |
| Chemotherapy |  |  | 0.173 |
| No | 170 (84.2%) | 376 (79.7%) |  |
| Yes | 32 (15.2%) | 96 (20.3%) |  |

AJCC: American Joint Committee on Cancer; NETs: neuroendocrine tumours; SEER: Surveillance, Epidemiology, and End Results; LN: lymph nodes
