# Supplementary material for: A modified mTNM staging system based on lymph node ratio for colon neuroendocrine tumors: A recursive partitioning analysis
Source: Front Surg. 2022 Oct 21;9:961982. doi: 10.3389/fsurg.2022.961982 (PMC9634476; doi:10.3389/fsurg.2022.961982)
Supplement: Supplementary file 2 [file Table2.docx]

**Supplementary Table S2.** The distinguishing ability of each classification system

| **Variables** | **C-index** | **95%CI** | **p-value** |
| --- | --- | --- | --- |
| AJCC N classification | 0.568 | 0.554-0.582 | 1 |
| Modified N classification | 0.623 | 0.605-0.641 | <0.01 |
| AJCC I\II\III classification | 0.587 | 0.575-0.599 | 1 |
| modified I\II\III classification | 0.665 | 0.6480.682 | <0.01 |

CI, confidence interval; AJCC, American Joint Commission on Cancer
