# Supplementary figures and images for: A modified mTNM staging system based on lymph node ratio for colon neuroendocrine tumors: A recursive partitioning analysis
Source: Front Surg. 2022 Oct 21;9:961982. doi: 10.3389/fsurg.2022.961982 (PMC9634476; doi:10.3389/fsurg.2022.961982)

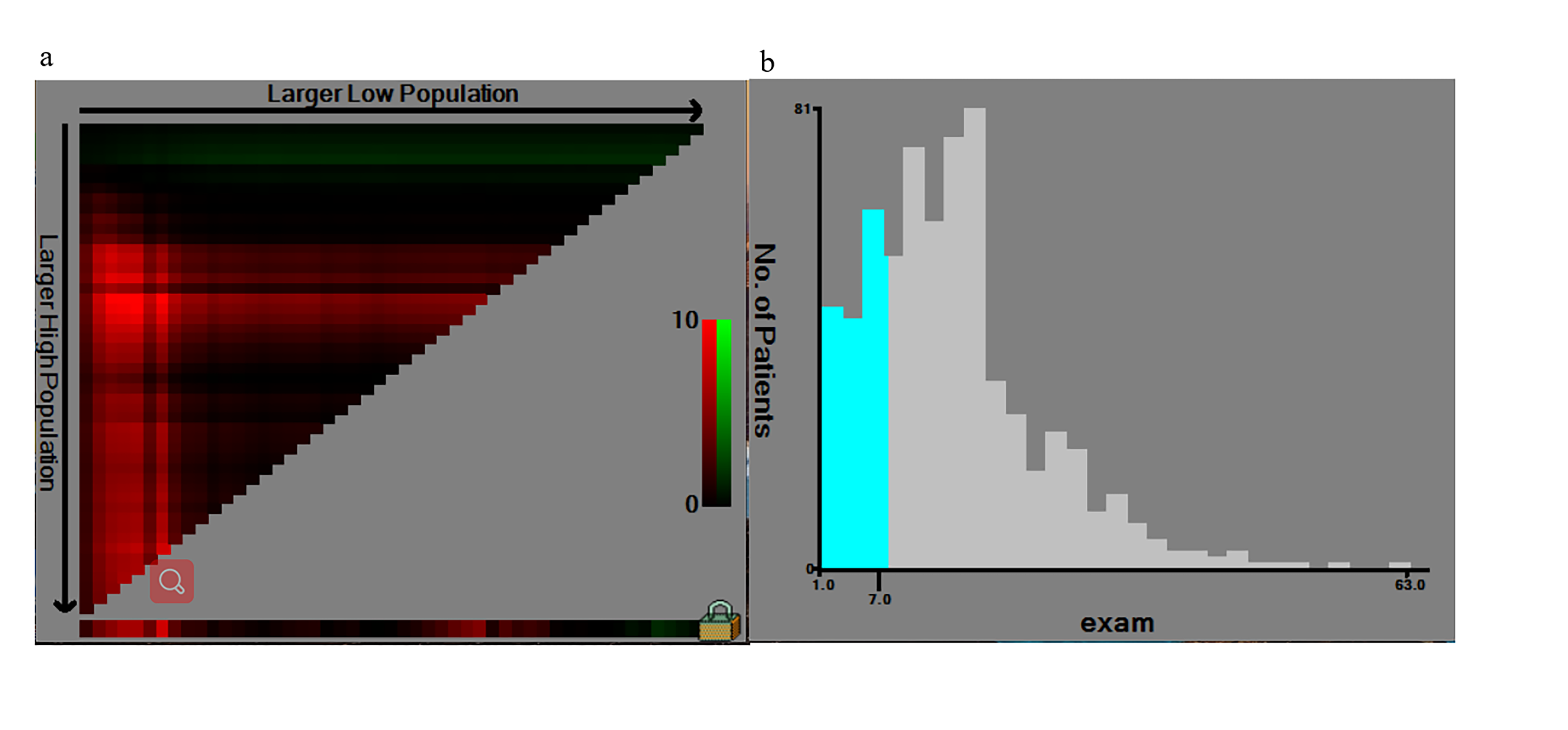

Supplement: Supplementary file 3 [file Image1.tif]
